# Deep learning can be used for accurate classification of hand osteoarthritis using plain radiographs

**DOI:** 10.1002/jeo2.70639

**Published:** 2026-03-25

**Authors:** Michael Axenhus, Jonas Havela, Anna Wallin, Sara Severin, Ablikim Karahan, Max Gordon, Martin Magnéli

**Affiliations:** ^1^ Department of Orthopaedic Surgery Danderyd University Hospital Stockholm Sweden; ^2^ Department of Clinical Sciences at Danderyd Hospital Karolinska Institutet Stockholm Sweden

**Keywords:** classification, convolutional neural networks, hand osteoarthritis, Kellgren–Lawrence, plain radiographs

## Abstract

**Purpose:**

Hand osteoarthritis poses a growing health concern, necessitating accurate and efficient diagnostic methods. Convolutional neural networks offer a promising approach for automating osteoarthritis classification, but their application to hand osteoarthritis remains underexplored. This study aimed to develop and evaluate a convolutional neural network model for detecting and classifying hand osteoarthritis on plain radiographs according to the Kellgren–Lawrence (KL) and modified Eaton–Littler (EL) classification systems.

**Methods:**

A total of 7515 examinations were retrospectively collected (27,965 images) from one hospital (2002–2016) and split into training (*n* = 7,100), validation (*n* = 415), and test (*n* = 327) sets with no patient overlap. Images were labelled for presence, grade, and joint involvement (interphalangeal, metacarpophalangeal, thumb carpometacarpal, and scaphotrapeziotrapezoidal joints). A residual‐network model with 39 convolutional layers was trained from scratch in PyTorch with data augmentation (random rotation, flipping, and cropping).

**Results:**

The convolutional neural network model achieved adequate performance in detecting hand OA (AUC = 0.91, sensitivity = 90%, specificity = 83%) and classifying osteoarthritis grades. However, lower osteoarthritis grades were challenging, particularly in the CMC1 and STT joints. Despite limitations in inter‐reader reliability of classification systems, the convolutional neural network model demonstrated capability in localising osteoarthritis findings.

**Conclusion:**

This study demonstrates the feasibility of convolutional neural network models in automating hand osteoarthritis detection and classification. The model's adequate performance suggests its potential for clinical use in enhancing classification accuracy and streamlining workflows. Challenges in lower‐grade osteoarthritis classification highlight the need for improved standardisation in classification systems. Further research is warranted to validate and refine the model's performance in diverse clinical settings and assess its real‐world implications.

**Level of Evidence:**

Level III.

## Graphical abstract

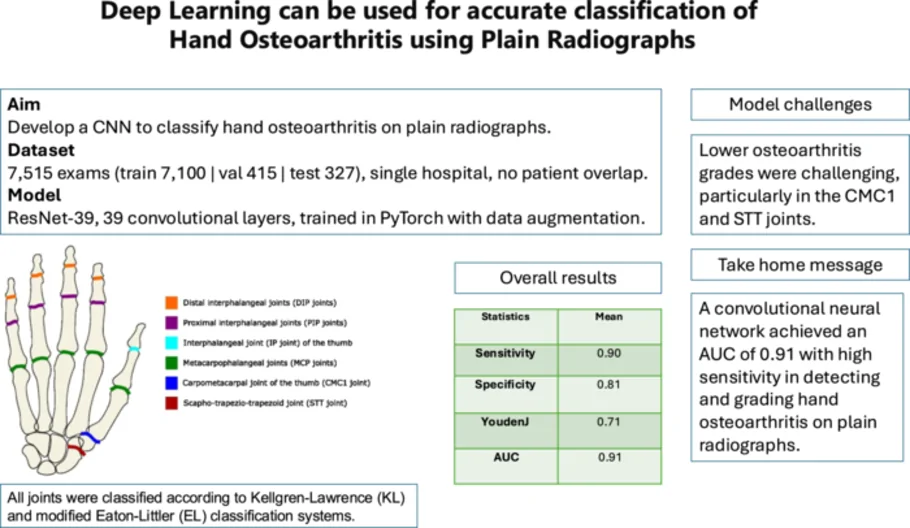

**Abbreviations**
AUC: area under the (ROC) curve
CI: confidence interval
CMC: carpometacarpal (joint)
CMC1/CMC‐I: first carpometacarpal joint (thumb base)
CNN: convolutional neural network
DIP: distal interphalangeal (joint)
EL: (modified) Eaton–Littler classification
ESCISIT: EULAR Standing Committee for International Clinical Studies Including Therapeutics
EULAR: European Alliance of Associations for Rheumatology (formerly European League Against Rheumatism)
IP: interphalangeal (joint)
J: Youden's index
KL: Kellgren–Lawrence (classification)
MCP: metacarpophalangeal (joint)
OA: osteoarthritis
OAI: osteoarthritis Initiative
PA: posteroanterior (projection)
PACS: Picture Archiving and Communication System
PIP: proximal interphalangeal (joint)
RA: rheumatoid arthritis
ResNet: residual network (CNN architecture)
ROC: receiver operating characteristic
SGD: stochastic gradient descent
STT: scaphotrapeziotrapezoidal (joint)

## INTRODUCTION

Hand osteoarthritis (OA) is one of the most common forms of OA [19]. While radiographic signs of hand OA are very common in the elderly, the age‐adjusted prevalence of symptomatic hand OA is estimated between 2.0‐6.2% [10]. The prevalence is expected to rise in upcoming decades [13]. Symptomatic hand OA may vary from mild to severe disease manifestations [18]. Therapy‐resistant cases may be subject to surgical procedures, such as interposition arthroplasty, osteotomy or arthrodesis [32]. Hand OA represents a significant and growing burden on a global scale. Studies from countries such as India, China, and Brazil report comparable or even higher rates of symptomatic OA, often influenced by occupational load and limited access to early diagnosis and treatment [1, 14, 22].

Hand OA is primarily a clinical diagnosis, however, plain radiography is often performed to determine OA‐related findings [25]. Radiographic OA grade is often associated with the degree of hand pain and function [5]. Various classification systems for radiographic hand OA exist, with the Kellgren–Lawrence (KL) classification being most common [30]. The KL classification grades OA findings on a scale from 0 to 4 [30]. Thumb base OA is most frequently classified using the modified Eaton‐Littler (EL) classification, also considering the degree of CMC1‐joint subluxation [9].

Convolutional neuronal networks (CNN) have emerged as a potential tool to detect and grade OA with studies showing excellent accuracy in knee, hip and shoulder OA [12, 17]. A few studies have used CNN to differentiate between normal findings, OA, and rheumatoid arthritis in the hand from plain radiographs while achieving high accuracies [15, 29]. Other studies have focused on training CNNs to measure specific OA‐related findings in the hand joints, such as joint space narrowing [24]. However, no study has utilised CNN to classify of hand OA grade or detect specific affected joints.

The aim of this study was to train and evaluate a CNN model to identify and classify hand OA in the IP, MCP, CMC and STT joints, according to the KL and modified EL classification systems, on plain radiographs. It was hypothesised that the model would accurately identify affected joints and grade disease severity with diagnostic performance comparable to human readers.

## MATERIALS AND METHODS

### Study design and sample

In this retrospective study, a total of 7515 comprising 27,965 images in a population aged 15 years or older were obtained from the Picture Archiving and Communication System (PACS) at Danderyd University Hospital. The images were all obtained between 2002 and 2016 for clinical purposes. Each examination contained 2–7 radiographs and variability in the number of radiographs may result in uneven patient representation. To minimise bias in the model training structure, no patient appeared in more than one of the training, validation, or test sets. All patient data were removed during the retrieval process. All wrist and hand radiographs were included, and no exception was made regarding radiographs containing implants and casts. Examinations of patients younger than 15 years of age were excluded.

### Datasets

The examinations were distributed between the training set (*n* = 7100), validation set (*n* = 415) and test set (*n* = 327). There was no patient overlap between the datasets. The distribution of desired pathologies was aimed to be proportional across the training and validation sets. For the training dataset, examinations were initially randomly selected from the PACS. Thereafter, radiologists' reports were searched for keywords in order to find examinations with hand OA in categories with low prevalence in the training set. After intermittent testing against the validation dataset, further keyword searches were done to find examinations in categories with poor model performance. For the validation dataset, examinations were selected through keyword searches to find examinations with either hand OA or normal findings. Retrieval and labelling of new examinations for the training dataset continued until intermittent testing ceased to reveal increased model performance. For the validation dataset, the process continued until OA categories had an adequate number of cases for statistical analyses. Two experienced senior orthopaedic surgeons, blinded to the network's predictions and initially independent of each other, retrieved and classified the test set. Images with differing classifications were revisited, and consensus was reached after mutual discussion. The same patient could appear multiple times when examinations were performed 90 days apart, but there was no overlap between the training, validation, and testing sets. The radiographs used were anonymized and did not contain any patient data.

### Outcome variables

Classification of hand OA in the IP joints, MCP joints, CMC1 joint and STT joint (Figure 1). The classification was done according to the KL and modified EL classification systems.

**Figure 1:**
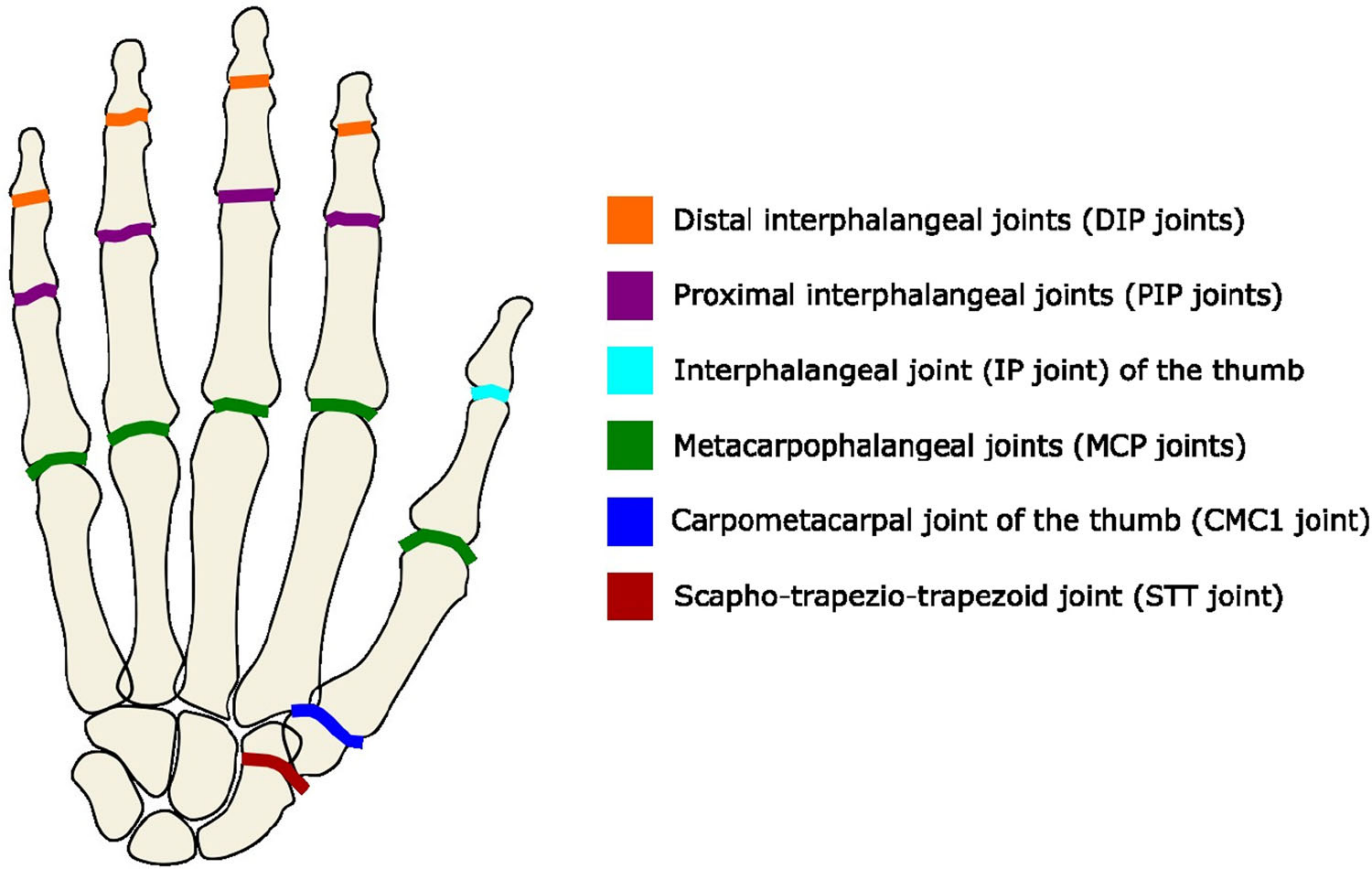
Overview of hand joints commonly affected by hand osteoarthritis.

### Review and labelling process

The retrieved examinations were uploaded to an in‐house developed online labelling platform where tools to label plain radiographic images based on KL and EL classification systems were available. Original radiologist's reports were included in most cases. Two fourth‐year medical students from Karolinska Institutet, Stockholm, Sweden, labelled the training and validation sets under the supervision of two senior orthopaedic surgeons. The students were trained in labelling the radiographs with direct supervision in the first stages. After becoming independent in labelling a series of validation and consensus sessions were completed. The labels served as a ground truth. Examinations in the training set were labelled only once, while examinations in the validation set were labelled separately by each medical student. Difficult cases where correct labelling was uncertain were revisited under supervision by a senior orthopaedic surgeon. Additionally, examinations in the validation set with differing labels between the students were reassessed under supervision until a consensus was reached.

As network training was initiated, the network gradually learned to predict labels for each examination during subsequent labelling. The network's predictions were incorporated into the online labelling platform and were presented to the human observers as a degree of network certainty ranging between <50% certainty, 50%–70% certainty or >70% certainty of selected labels. The human observers had the choice of keeping the network‐predicted labels for the particular study or changing the labels based on their own assessment of the study.

Each examination was analysed and labelled for radiographic findings of OA in the IP joints, the MCP joints, the CMC1 joint, and the STT joint. When OA was present, it was graded according to the KL classification in the IP joints and MCP joints, and according to the modified EL classification in the CMC1 joint and STT joint. If consensus on an OA grade could not be reached, the grade was recorded as a range of two possible categories (e.g., ‘OA grade II or III’). All examinations were also labelled for other forms of arthritis, fractures, osteopenia, implants, casts, open physes, as well as a category for other pathology not mentioned above.

The most common hand OA grade throughout the hand joints was IV (Figure 2).

**Figure 2:**
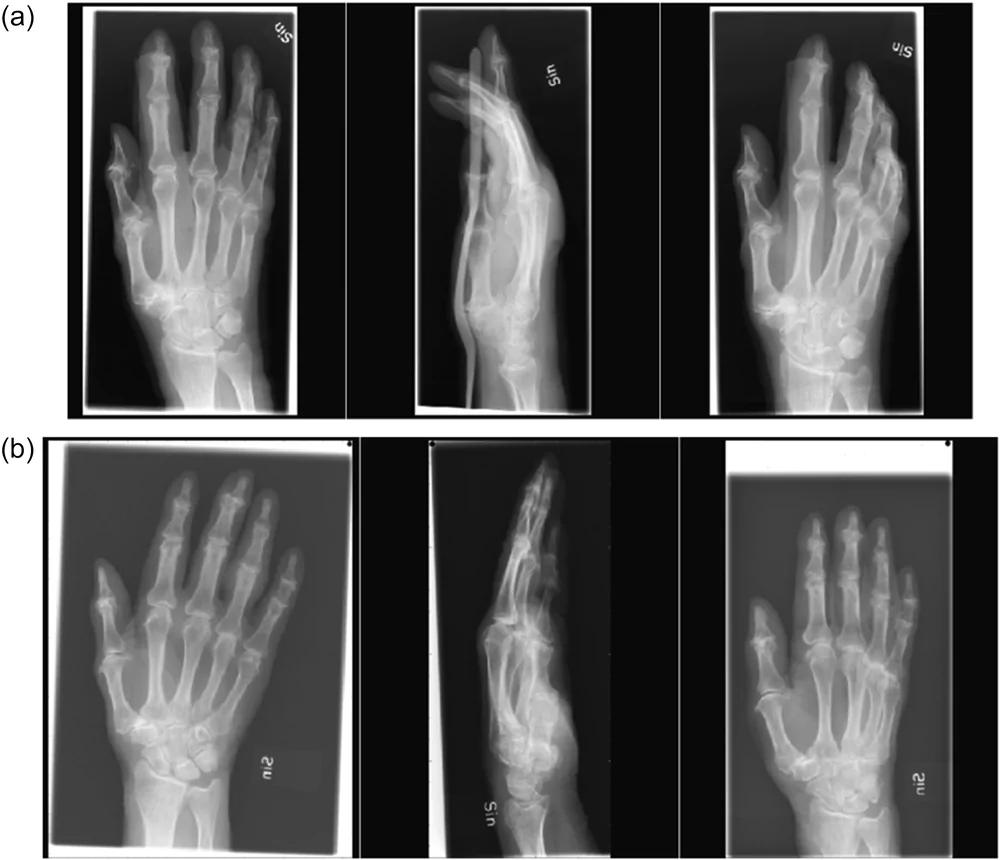
Examples of OA grade correctly and incorrectly graded by the network. In this case the CNN graded OA grade IV (a) correctly and OA grade III (b) incorrectly.

The highest prevalence of severe OA (Grade IV) is observed in the CMC‐I and STT joints, with 47 cases (14.4%) in the test set for both joints. Grade I OA is relatively uncommon across most joints, with as few as 0.6% for certain categories like STT and CMC‐I (Table 1a). The DIP and PIP joints also show considerable OA involvement, particularly in the DIP I‐IV joints (Table 1b). Severe OA was most prevalent in the first metacarpal joint, whereas lower grades were less frequent across the remaining metacarpals (Table 1c).

**Table 1a:** The distribution of OA in the joints of the thumb joints in the test, validation and training sets.

Joint	Grade	Test (*n* = 327) *n* (%)	Validation (*n *= 415) *n* (%)	Train (*n* = 7100) *n* (%)
STT	I	2 (0.6)	4 (1.0)	111 (1.6)
	II	9 (2.8)	7 (1.7)	122 (1.7)
	III	5 (1.5)	3 (0.7)	48 (0.7)
	IV	47 (14.4)	38 (9.2)	418 (5.9)
CMC‐I	I	3 (0.9)	5 (1.2)	121 (1.7)
	II	17 (5.2)	14 (3.4)	227 (3.2)
	III	11 (3.4)A	23 (5.5)	196 (2.8)
	IV	47 (14.4)	38 (9.2)	417 (5.9)

*Note*: Roman numerals indicate OA grade.

Abbreviations: CMC‐I, first carpometacarpal; STT, scaphotrapeziotrapezoidal.

**Table 1b:** The distribution of OA in the joints of the fingers and thumb in the test, validation and training sets.

Joint/digit	Grade	Test *n* (%)	Validation *n* (%)	Train *n* (%)
Digit 1 – interphalangeal (IP)	I	8 (2.4)	18 (4.3)	241 (3.4)
	II	27 (8.3)	16 (3.9)	173 (2.4)
	III	14 (4.3)	5 (1.2)	83 (1.2)
	IV	4 (1.2)	6 (1.4)	26 (0.4)
Digit 2 – PIP	I	8 (2.4)	17 (4.1)	145 (2.0)
	II	7 (2.1)	7 (1.7)	76 (1.1)
	III	4 (1.2)	5 (1.2)	40 (0.6)
	IV	3 (0.9)	4 (1.0)	41 (0.6)
Digit 2 – DIP	I	9 (2.8)	20 (4.8)	257 (3.6)
	II	12 (3.7)	14 (3.4)	214 (3.0)
	III	14 (4.3)	11 (2.7)	92 (1.3)
	IV	8 (2.4)	14 (3.4)	93 (1.3)
Digit 3 – PIP	I	6 (1.8)	18 (4.3)	166 (2.3)
	II	14 (4.3)	7 (1.7)	77 (1.1)
	III	1 (0.3)	4 (1.0)	35 (0.5)
	IV	6 (1.8)	5 (1.2)	41 (0.6)
Digit 3 – DIP	I	11 (3.4)	22 (5.3)	274 (3.9)
	II	13 (4.0)	11 (2.7)	165 (2.3)
	III	9 (2.8)	16 (3.9)	94 (1.3)
	IV	12 (3.7)	12 (2.9)	73 (1.0)
Digit 4 – PIP	I	4 (1.2)	21 (5.1)	173 (2.4)
	II	14 (4.3)	9 (2.2)	64 (0.9)
	III	2 (0.6)	2 (0.5)	22 (0.3)
	IV	6 (1.8)	5 (1.2)	44 (0.6)
Digit 4 – DIP	I	12 (3.7)	26 (6.3)	258 (3.6)
	II	11 (3.4)	11 (2.7)	144 (2.0)
	III	6 (1.8)	10 (2.4)	61 (0.9)
	IV	6 (1.8)	6 (1.4)	37 (0.5)
Digit 5 – PIP	I	10 (3.1)	15 (3.6)	169 (2.4)
	II	12 (3.7)	6 (1.4)	83 (1.2)
	III	2 (0.6)	3 (0.7)	38 (0.5)
	IV	6 (1.8)	6 (1.4)	34 (0.5)
Digit 5 – DIP	I	11 (3.4)	20 (4.8)	271 (3.8)
	II	17 (5.2)	17 (4.1)	175 (2.5)
	III	10 (3.1)	9 (2.2)	102 (1.4)
	IV	6 (1.8)	12 (2.9)	45 (0.6)

*Note*: Roman numerals indicate OA grade.

Abbreviations: CMC‐I, first carpometacarpal; STT, scaphotrapeziotrapezoidal.

**Table 1c:** The distribution of OA in the metacarpal joints in the test, validation and training sets.

Joint	Grade	Test *n* (%)	Validation *n* (%)	Train *n* (%)
Metacarpal 1	I	3 (0.9)	14 (3.4)	99 (1.4)
	II	17 (5.2)	2 (0.5)	63 (0.9)
	III	11 (3.4)	3 (0.7)	35 (0.5)
	IV	47 (14.1)	4 (1.0)	16 (0.2)
Metacarpal 2	I	2 (0.6)	1 (0.2)	45 (0.6)
	II	2 (0.6)	3 (0.7)	47 (0.7)
	III	2 (0.6)	2 (0.5)	21 (0.3)
	IV	6 (1.8)	1 (0.2)	13 (0.2)
Metacarpal 3	I	6 (1.8)	5 (1.2)	54 (0.8)
	II	4 (1.2)	1 (0.2)	29 (0.4)
	IV	2 (0.6)	1 (0.2)	13 (0.2)
Metacarpal 4	I	3 (0.9)	3 (0.7)	31 (0.4)
	II	1 (0.3)	2 (0.5)	24 (0.3)
Metacarpal 5	I or II	13 (4.0)	10 (2.4)	146 (2.1)
	III or IV	9 (2.8)	5 (1.2)	54 (0.8)

*Note*: Roman numerals indicate OA grade.

Abbreviation: Metacarpal, metacarpophalangeal joint.

### Model architecture and model training

The study utilised the open‐source machine learning framework PyTorch (v 2.0). A CNN of ResNet architecture consisting of 39 convolutional layers was used, each layer equipped with batch normalisation, and an adaptive max pool [8]. The network was randomly initiated and trained using stochastic gradient descent. The training dataset labels served as ground truth during training, and examinations were processed by the network for 80 epochs. To increase the robustness of the training, each image was also rotated, flipped and randomly cropped. The final CNN setup used for testing the model against validation dataset label (Table 2).

**Table 2:** Neural network setup.

Type	Blocks	Kernel size	Filters	Group
ResNet block	1	3×3	64	Image
ResNet block	1	3×3	64	Image
ResNet block	2	3×3	64	Core
ResNet block	2	3×3	128	Core
ResNet block	6	3×3	256	Core
ResNet block	6	3×3	512	Core
Average for top	1	‐	‐	Pool
50% views				
Convolutional	1	1×1	72	Classification
Fully connected	1	1×1	4	Classification
Fully connected	1	1×1	4	Classification

### Statistics

Statistical analysis was performed with the software program R (v 4.3.0). Model performance was assessed using sensitivity, specificity, Youden's index (J), and area under the receiver operating characteristics (ROC) curve (AUC) [31]. For the AUC value, a 95% confidence interval (CI) was analysed. AUC was used as outcome measure as described by Mandrekar et al., where an AUC of 0.5 is considered no better than pure chance, 0.7–0.8 is acceptable, 0.8–0.9 is excellent, whereas values over 0.9 are considered outstanding [16]. A ROC curve is a plot with sensitivity on the *Y*‐axis, versus 1‐specificity on the *X*‐axis, and is a tool for assessing the performance of diagnostic tests, taking into account the tests performance at different thresholds [4, 20].

### Ethics

This study did not need an ethical permit due to the use of deidentified images. This decision was established by the Swedish Ethical Review Authority (2014/453‐31).

## RESULTS

### Thumb osteoarthritis

The test dataset contained 78 cases of CMC‐I joint OA and 63 cases of STT joint OA. Higher grades of OA generally exhibit higher sensitivity and specificity, with EL Grade IV having the highest sensitivity at 100% in the CMC‐I and 92% in the STT joint. Lower OA grades in the CMC and STT joint were harder to classify compared to other joints and grades (Table 3).

**Table 3:** Model performance in identifying and classifying OA in thumb joints.

Joint and OA grade	Cases (n = 327)	Sensitivity (%)	Specificity (%)	Youden's J	AUC (95% CI)
CMC‐1					
I	3	100	52	0.52	0.67 (0.51 to 0.83)
II	17	100	64	0.64	0.86 (0.79 to 0.92)
III	11	91	72	0.62	0.82 (0.73 to 0.91)
IV	47	89	84	0.73	0.93 (0.90 to 0.96)
IP					
I	8	75	64	0.39	0.68 (0.51 to 0.85)
II	27	96	72	0.69	0.85 (0.79 to 0.91)
III	14	93	93	0.86	0.95 (0.90 to 1.00)
IV	4	100	82	0.82	0.91 (0.84 to 0.98)
MCP 1					
I	3	100	52	0.52	0.67 (0.51 to 0.83)
II	17	100	64	0.64	0.86 (0.79 to 0.92)
III	11	91	72	0.62	0.82 (0.73 to 0.91)
IV	47	89	84	0.73	0.93 (0.90 to 0.96)
STT					
I	2	100	67	0.67	0.77 (0.57 to 0.98)
II	9	100	39	0.39	0.68 (0.55 to 0.82)
III	5	100	62	0.62	0.76 (0.64 to 0.87)
IV	47	87	85	0.72	0.93 (0.90 to 0.96)

*Note*: Roman numerals indicate OA grade.

### Finger osteoarthritis

The CNN exhibited high sensitivity, reaching 100% across many joint groups and OA grades in the fingers, particularly in the more severe grades (III and IV). However, while sensitivity remained high across the board, specificity was more variable, particularly for lower OA grades. Specificity was lower for grades such as MCP 2 (Grade II) and DIP 3 (Grade II), where it dropped to 62% (Table 4).

**Table 4:** Model performance in identifying and classifying OA in finger joints.

Joint and OA grade	Cases (*n* = 327)	Sensitivity (%)	Specificity (%)	Youden's J	AUC (95% CI)
DIP 2					
I	9	100	74	0.74	0.87 (0.81 to 0.94)
II	12	92	80	0.71	0.88 (0.82 to 0.94)
III	14	86	88	0.74	0.90 (0.83 to 0.97)
IV	8	100	77	0.77	0.95 (0.89 to 1.00)
MCP 2					
I	2	100	84	0.84	0.92 (0.77 to 1.00)
II	2	100	62	0.62	0.72 (0.52 to 0.92)
III	2	100	70	0.70	0.85 (0.55 to 1.00)
IV	6	100	90	0.90	0.96 (0.92 to 0.99)
PIP 2					
I	8	100	72	0.72	0.87 (0.79 to 0.94)
II	7	100	89	0.89	0.94 (0.91 to 0.98)
III	4	100	88	0.88	0.92 (0.88 to 0.97)
IV	3	100	74	0.74	0.91 (0.74 to 1.00)
DIP 3					
I	11	100	68	0.68	0.87 (0.80 to 0.94)
II	13	85	62	0.47	0.75 (0.63 to 0.87)
III	9	100	84	0.84	0.94 (0.90 to 0.98)
IV	12	100	86	0.86	0.95 (0.92 to 0.98)
MCP 3					
I	6	83	94	0.77	0.93 (0.85 to 1.00)
II	4	100	68	0.68	0.82 (0.70 to 0.94)
IV	2	50	97	0.47	0.57 (0.00 to 1.00)
PIP 3					
I	6	100	75	0.75	0.89 (0.82 to 0.97)
II	14	100	90	0.90	0.96 (0.93 to 0.98)
IV	6	100	83	0.83	0.94 (0.89 to 0.99)
DIP 4					
I	12	100	65	0.65	0.85 (0.78 to 0.93)
II	11	100	67	0.67	0.87 (0.80 to 0.93)
III	6	100	92	0.92	0.96 (0.93 to 0.99)
IV	6	100	88	0.88	0.96 (0.92 to 1.00)
MCP 4					
I	3	100	90	0.90	0.95 (0.89 to 1.00)
PIP 4					
I	4	100	73	0.73	0.88 (0.76 to 1.00)
II	14	93	88	0.81	0.91 (0.87 to 0.95)
III	2	100	95	0.95	0.96 (0.93 to 1.00)
IV	6	100	90	0.90	0.97 (0.94 to 1.00)
DIP 5					
I	11	91	87	0.78	0.89 (0.85 to 0.94)
II	17	100	70	0.70	0.91 (0.86 to 0.96)
III	10	100	83	0.83	0.94 (0.90 to 0.97)
IV	6	100	93	0.93	0.97 (0.94 to 0.99)
MCP 5					
II	3	100	95	0.95	0.98 (0.95 to 1.00)
PIP 5					
I	10	90	80	0.70	0.90 (0.83 to 0.97)
II	12	100	79	0.79	0.92 (0.87 to 0.96)
III	2	100	95	0.95	0.96 (0.93 to 0.98)
IV	6	100	94	0.94	0.99 (0.97 to 1.00)

*Note*: Roman numerals indicate OA grade.

### Overall performance

Mean summary weighted statistics of the test set showed an AUC of 91% and a sensitivity of 90% (Table 5).

**Table 5:** Mean weighted summary statistics of the test dataset.

Statistics	Mean
Sensitivity	0.90
Specificity	0.81
YoudenJ	0.71

*Note*: Each analysis is multiplied by the number of cases and then divided by the total case number.

## DISCUSSION

Numerous studies have highlighted the capability of utilising CNN models for OA detection and classification, with knee and hip OA receiving the most attention [2]. The purpose of this study was to investigate whether a CNN model could be developed to detect and classify hand OA from plain radiographs. Overall, the CNN model performed with an AUC of 0.91 in detecting findings of OA, which is a promising result for automating OA identification in hands [27]. Additionally, in most categories, the model performed with high AUC values in detection and classifying of OA severity grade. However, lower OA grades were more challenging, especially in the CMC1 joint and STT joint which mirrors clinical practice.

Two previous studies have examined the application of CNN models for the detection of hand OA. First, Üreten et al. utilised a pre‐trained CNN in order to differentiate hand OA, rheumatoid arthritis, and normal findings on plain radiographs, including 1428 examinations with only one posteroanterior image per examination [29]. Their model achieved an AUC of 0.96 in distinguishing hand OA from normal findings. Second, Ma et al. trained and developed a CNN model with a total of 9714 examinations, including frontal, oblique and lateral views, achieving an AUC of 0.97 in discriminating between hand OA and normal findings [15]. In line with these two studies, the CNN model effectively detected OA findings in the hand.

Some major differences exist between this study and the studies by Ureten et al. and Ma et al. In contrast, the current study used all images included in the examinations from the PACS to both train and validate the model. Furthermore, the aim was to develop a CNN model capable of grading severity of hand OA and discerning the precise joints affected. To our knowledge, this study represents the first attempt to develop a CNN model specifically for detecting hand OA while also specifying and grading each affected joint.

In a notable study from 2018, Tiulpin et al. developed a CNN model to both diagnose as well as grade knee OA according to the KL classification system from plain radiographs, achieving a multi‐class accuracy of 67% in correctly assigning the KL grade [28]. Similarly, Swiecicki et al. developed a CNN model, reaching high accuracies in classifying knee OA according to the KL system [27]. Consistent with these studies, the current CNN model was able to grade hand OA severity with high accuracy.

The CNN model performed proficiently not only in detecting and classifying hand OA in general, but also in specifying the exact joint affected. Localising OA from plain radiographs, Olsson et al. utilised a CNN model to differentiate between knee OA affecting the lateral or medial joint space, as well as patellofemoral OA and achieving high accuracy [21]. While the CNN had adequate performance in localising OA findings, the results should also be considered against the complex anatomy of the hand, with a total of 16 joint spaces per hand trained and evaluated in this study.

Low specificity in lower grades of OA could be explained by the subjectivity of the OA classification systems. Swiecicki et al. found only moderate agreement between radiologists in grading knee OA according to the KL classification, especially for lower KL grades. Regarding the modified EL classification system [27]. Spaans et al. found only moderate inter‐observer reliability regarding OA in the CMC1 joint [26]. The subjectivity inherent in these classification systems likely contributed to inter‐reader variability during the labelling process, potentially impacting the CNN model's learning. Since higher classification grades have more obvious radiographic findings, labelling and thus CNN learning may have been more consistent resulting in better performance. Moreover, the modified EL classification system is especially ambiguous in its grade 1 description, which could explain the challenges in classifying lower grades of CMC1 joint and STT joint OA. Grade 1 is defined as subtle carpometacarpal joint space widening, thought to represent an early synovitis phase of the disease [9]. Interpreting subtle joint space widening is indeed difficult, especially considering the large variation of projections included in the study. Furthermore, when applying the modified EL system in labelling the STT joint, the grade 1 definition with joint space widening is not applicable to the STT joint. Thus, ambiguity in classifying grade 1 OA in both the CMC1 joint and the STT joint may explain the CNN model's difficulties in interpreting lower grades of OA. Another explanation for the variance in accuracy in the model could be the presence of class imbalances across the different OA grades and joints, with more severe OA grades (III and IV) being more prevalent in certain joints like the CMC‐I and DIP joints. These imbalances may have impacted the model's performance, particularly in the less represented categories, potentially leading to reduced accuracy for early‐stage OA classification.

A major strength of this particular approach lies in the large dataset. In a systematic review by Binvignat et al. who described machine learning in OA, the mean number of examinations was 1,359, significantly less than in the current study [2]. Another strength is the diversity of projections included in each examination. Extracted from the PACS of Danderyd Hospital, all images in each examination were included, regardless of image characteristics such as the presence of implants or casts, or suboptimal projections, reflecting clinical conditions which in turn increase internal validity. This approach contrasts to most other studies in the field of deep learning and OA. Binvignat et al. noted that a majority of studies used imaging data from standardised databases such as the Osteoarthritis Initiative which contain images taken in a standardised way solely for the purpose of involvement in studies [7].

This study has several limitations. Firstly, examinations were labelled by two fourth‐year medical students. Chen et al. found individual medical novices performing at radiologists' level in diagnosing hip fractures from plain radiographs after only limited training, nevertheless, they were outperformed by the radiology professionals as a group [3]. As discussed by Choi et al., since deep learning models rely on training data to learn, incorrect or mislabelled examinations can distort the model's validity and real‐world applicability [4]. To mitigate this, initial labelling was done under supervision by an orthopaedic surgeon, and difficult cases were revisited under supervision. Secondly, the KL and EL classification systems have some inherent problems with intra‐reader and inter‐reader reliability. Many more criticisms have been raised, such as by Kohn et al. on the KL classification systems dependence on osteophyte formation [11]. Since the severity of radiographic hand OA findings have been correlated to the degree of symptoms [8, 14] [5, 23]. Lastly, it would be of interest to externally validate the model with data from other populations, to assess the model's generalisability. For the purposes of this study, only one type of neural network was used. Potentially, other designs of neural network could perform better at detecting and classifying hand OA.

This study demonstrates the feasibility of developing and training CNN model to detect and classify hand OA in specific joints. Furthermore, plain radiographic examinations are often double‐read by two radiologists. Dembrower et al. found replacing one radiologist with a deep learning model compared to radiologist double reading to be non‐inferior in the case of interpreting screening mammograms [6]. Integrating such models into radiology workflows could improve diagnostic efficiency and grading consistency. Future work should focus on external validation in diverse populations to ensure generalisability, including settings with limited access to expert radiological assessment. Prospective studies are also warranted to evaluate the ability for CNN models to monitor disease progression and support clinical decisions.

## CONCLUSIONS

In conclusion, this study demonstrated that a CNN can accurately detect and classify hand osteoarthritis on plain radiographs according to the KL and modified EL grading systems. The model performed well in identifying affected joints and grading advanced disease, although lower grades, particularly in the thumb and STT joints remained more difficult to classify.

Based on these findings, further development and external validation of CNN models are warranted. Integration of such tools into radiology workflows could improve diagnostic consistency and efficiency. Future longitudinal studies should also explore their role in monitoring disease progression and supporting clinical decisions.

## AUTHOR CONTRIBUTIONS

*Conceptualisation*: Max Gordon and Martin Magnéli. *Methodology*: Max Gordon and Martin Magnéli. *Software*: Michael Axenhus, Max Gordon and Martin Magnéli. *Validation*: Max Gordon and Martin Magnéli. *Formal analysis*: Jonas Havela, Anna Wallin and Martin Magnéli. *Investigation*: Jonas Havela, Anna Wallin, Martin Magnéli and Ablikim Karahan. *Resources*: Max Gordon and Martin Magnéli. *Data curation*: Max Gordon and Martin Magnéli. *Writing‐original draft*: Michael Axenhus. *Writing‐review & editing*: Max Gordon, Martin Magnéli, Ablikim Karahan, Sara Severin, Anna Wallin and Jonas Havela. *Visualisation*: Michael Axenhus, Jonas Havela and Martin Magnéli. *Supervision*: Michael Axenhus, Max Gordon and Martin Magnéli.

## CONFLICT OF INTEREST STATEMENT

The authors declare that they have no competing interests.

## ETHICS STATEMENT

An ethical permit has been granted by the Ethical Review Board in Stockholm, Sweden (Dnr 2014/453‐31/3).

## Data Availability

The raw datasets are available from the corresponding author on reasonable request.
